# E-cigarette puffing patterns associated with high and low nicotine e-liquid strength: effects on toxicant and carcinogen exposure

**DOI:** 10.1186/s12889-016-3653-1

**Published:** 2016-09-20

**Authors:** Sharon Cox, Leon Kośmider, Hayden McRobbie, Maciej Goniewicz, Catherine Kimber, Mira Doig, Lynne Dawkins

**Affiliations:** 1 London South Bank University, Division of Psychology, School of Applied Sciences, 103 Borough Rd, London, UK; 2 Institute of Occupational Medicine and Environmental Health, Koscielna 13 Street, Sosnowiec, Poland; 3 Queen Mary University of London, Wolfson Institute of Preventive Medicine, Barts and The London School of Medicine and Dentistry, Charterhouse Square, London, UK; 4 Roswell Park Cancer Institute, Department of Health Behavior, Elm and Carlton Streets, Buffalo, NY 14263 USA; 5 University of East London, School of Psychology, Waters Lane, London, UK; 6 ABS Laboratories Ltd, BioPark, Broadwater Road, Welwyn Garden City, Hertforshire AL7 3AX UK

**Keywords:** Electronic-cigarette, ENDS, E-cigarettes, Nicotine, Puffing patterns, Puffing topography, E-liquids, Toxicants, Carcinogens

## Abstract

**Background:**

Contrary to intuition, use of lower strength nicotine e-liquids might not offer reduced health risk if compensatory puffing behaviour occurs. Compensatory puffing (e.g. more frequent, longer puffs) or user behaviour (increasing the wattage) can lead to higher temperatures at which glycerine and propylene glycol (solvents used in e-liquids) undergo decomposition to carbonyl compounds, including the carcinogens formaldehyde and acetaldehyde. This study aims to document puffing patterns and user behaviour associated with using high and low strength nicotine e-liquid and associated toxicant/carcinogen exposure in experienced e-cigarette users (known as vapers herein).

**Methods/design:**

A counterbalanced repeated measures design. *Participants:* Non-tobacco smoking vapers; have used an e-cigarette for ≥3 months; currently using nicotine strength e-liquid ≥12mg/mL and a second or third generation device. *Intervention:* This study will measure puffing patterns in vapers whilst they use high and low strength nicotine e-liquid under fixed and user-defined settings, each for a week. The 4 counterbalanced conditions are: i) low strength (6mg/mL), fixed settings; ii) low strength user-defined settings; iii) high strength (18mg/mL) fixed settings; iv) high strength user-defined settings. Biomarkers of exposure to toxicants and carcinogens will be measured in urine. In the second phase of this study, toxicant yields will be measured in aerosol generated using a smoking machine operated to replicate the puffing behaviours of each participant. *Primary outcomes:* i) Puffing patterns (mean puff number, puff duration, inter-puff interval and mL of liquid consumed) and user behaviour (changes to device settings: voltage and air-flow) associated with using high and low strength nicotine e-liquid. ii) Toxicant/carcinogen exposure associated with the puffing patterns/device settings used by our participants. *Secondary outcomes:* i) Subjective effects. ii) comparisons with toxicant exposure from tobacco smoke (using documented evidence) and with recommended safety limits. *Sample size:* Twenty participants.

**Discussion:**

The findings will have important implications for public health messaging regarding the relative risks and subjective effects associated with using high and low strength nicotine e-liquid, and for policy makers regarding regulations on nicotine concentrations in e-liquids.

## Background

Electronic cigarette (known as e-cigarettes, ENDS and e-cigs) use is becoming increasingly popular among smokers wishing to quit or reduce smoking [1]. The rise in popularity may in part be driven by consumer demand for a less harmful alternative to smoking tobacco and/or a desire to reduce nicotine intake. Indeed, the often flavoured e-liquid offers a range of nicotine strengths from 0 % to 2.0 % = 20 mg/mL (since the implementation of the EU Tobacco Products Directive), thus providing a new way for users to monitor and reduce their nicotine intake in a less ambiguous manner than traditional cigarettes.

Smokers and vapers may opt for, or switch to, lower nicotine strength e-liquid for a variety of reasons: the belief that it is healthier; desire to wean off nicotine; or due to the EU Tobacco Products Directive which stipulates an upper limit of 20mg/mL nicotine concentrations in e-liquids introduced in May 2016. However, contrary to intuition, use of lower strength nicotine e-liquids might not offer reduced health risk if compensatory puffing behaviour occurs.

Compensatory puffing behaviour is well documented in tobacco smokers. Smokers increase puff frequency, duration and volume when switching to a lower nicotine yield cigarette [2–4] whilst maintaining high blood nicotine levels [5, 6]. Exposure to tar in smoke known to contain carcinogenic compounds is consequently increased [2, 7]. Compared with vapers, smokers typically take shorter puffs [8] and after switching to vaping, can adjust their puffing patterns within a week [8, 9]. Two small scale laboratory studies suggest that vapers can also compensate for lower nicotine strength e-liquid by altering their puffing patterns. Ramôa and colleagues [10], using a ten-puff protocol, reported that vapers took larger and deeper puffs with 0mg/mL vs. 36mg/mL nicotine strength e-liquid, which the authors concluded may reflect an attempt to self-titrate due to the absence of nicotine. Similarly, we Dawkins et al. [11] observed compensatory puffing patterns in which vapers took longer, more frequent puffs, and doubled the amount of e-liquid consumed during a one-hour *ad lib* puffing period with a low (6mg/mL) compared with a high (24mg/mL) strength nicotine e-liquid. In this study the device settings (voltage, wattage, resistance of the coil and air-flow) were fixed reflecting standard second-generation devices. In reality, experienced users may adjust these parameters when using lower nicotine strength e-liquid and this may in turn, influence toxicant/carcinogen exposure. In the user-defined condition proposed here, we will allow users to adjust the voltage and air-flow of the device (although atomiser resistance will be kept constant).

Exposure to toxicants and carcinogens from e-cigarettes is 9–450 times lower than from tobacco cigarettes [12], however, vaping is not risk-free and toxicant exposure will be related to the amount of e-liquid consumed. Longer, more frequent puffs and higher power may also lead to higher temperatures at which glycerine and propylene glycol, (solvents used in e-liquids), undergo decomposition to carbonyl compounds including the carcinogens formaldehyde and acetaldehyde [13]. If compensatory puffing patterns and changes to device settings lead to increased toxicant/carcinogen exposure due either to increased liquid consumption, increased temperature or both, switching to lower strength nicotine e-liquid may therefore not be the lower risk option.

We will measure puffing patterns in vapers in naturalistic conditions over four weeks whilst they use high and low strength nicotine e-liquid under fixed and user-defined (changes to device such as power output and air-flow allowed) settings, each for a week (4 conditions). Toxicant and carcinogen exposure will be assessed by measuring i) biomarkers of exposure in urine samples collected from participants and ii) yields of toxicants in e-cigarette aerosol generated for each individual under identical puffing conditions in the lab. Capturing e-cigarette puffing patterns and behaviour will not only inform the parameters of toxicity of the product used in the study but will have wider applicability for other researchers exploring toxicants/carcinogens in aerosol by ensuring realistic puffing patterns are used. Indeed, although the effects of solvent and power level on toxicant/carcinogen exposure have been explored [13, 14], laboratory studies to date have tested all products using the same puffing regime (ranging between studies from 1.8 to 4 s puff duration with a 10 to 60s inter-puff interval [IPI]; [13–17]. Such standardised puffing regimes not only fails to capture potential puffing variation between devices [18] and individuals [19, 20], but does not account for compensatory puffing with different nicotine yields [10, 11].

Whilst puffing patterns differed across high and low nicotine strength e-liquid conditions in our previous study, nicotine craving and withdrawal symptoms did not differ [11] suggesting that compensatory puffing behaviour was effective at least in the short term under laboratory conditions. Nevertheless, there was considerable individual variation on subjective reporting and laboratory conditions may not produce reliable evidence with respect to subjective effects because these settings can elicit unrealistic puffing behaviour [21]. The study outlined here will use a larger sample under naturalistic conditions to determine if and how real life puffing patterns associated with the use of high and low strength nicotine e-liquids differentially affect withdrawal symptoms, satisfaction and nicotine intake. Understanding the risks of compensatory puffing will have important implications for public health messaging regarding the relative risks and subjective effects associated with using high and low strength nicotine e-liquid, and for policy makers regarding regulations on nicotine concentrations in e-liquids.

### Objectives

This Cancer Research UK (CRUK) funded project will investigate e-cigarette puffing patterns and user behaviour associated with high and low strength nicotine e-liquids and how this affects toxicant and carcinogen exposure. We aim to measure natural consumer puffing behaviour in phase one of the experiment and to replicate these patterns within a laboratory setting within phase two. Specifically, we present four objectives:

- To measure puffing patterns (mean number of puffs, mean puff duration, mean IPI and mL liquid consumed) and user behaviour-changes to device settings - voltage and air-flow where permitted-associated with using high and low strength nicotine e-liquid to ascertain if and how compensatory puffing behaviour occurs.
- To explore if and how puffing patterns/user behaviour associated with high and low strength nicotine e-liquids affect satisfaction, craving, withdrawal symptoms and nicotine intake as measured by cotinine level in saliva.
- To determine whether puffing patterns/user behaviours associated with using low vs. high strength nicotine e-liquid increases toxicant/carcinogen exposure as measured by i) biomarker levels in users’ urine and ii) toxicant yields in e-cigarette aerosol (as measured in a laboratory setting).
- To assess the risk of whether toxicant/carcinogen exposure levels associated with the puffing patterns/device settings used by our participants are at a level likely to affect human health by comparing these with those found in tobacco smoke and against recommended safety limits.

### Study design

A repeated measured, counterbalanced, prospective cohort study.

## Methods

### Study setting

**Fig. 1:**
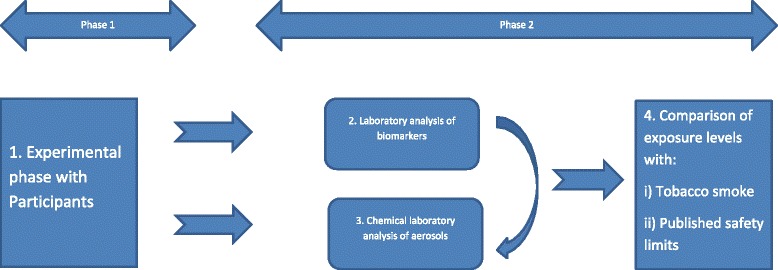
Illustration of Phase one and Phase two and respective stages

Figure 1 presents the study phases and stages.

#### Phase one

This study seeks to measure and replicate real-life puffing behaviours. Participants will receive minimal intervention in phase one of the study, only meeting with the research team on five occasions at baseline and at the end of each condition. Puffing data will be recorded by the e-cigarette device as participants complete their daily routines for the duration of phase one.

Puffing, biochemical and questionnaire data will be collected at London South Bank University within the School of Applied Sciences Psychology laboratories.

Urine and saliva samples will be sent off campus (see details below in Data collection) for analysis.

#### Phase two

Will replicate individual puffing topography from phase one, also at an external laboratory (see details below, in Data collection).

### Eligibility criteria

Participants will be eligible to take part providing they are aged 18 or over, an ex-smoker (reporting having not smoked at all for at least 3 months, confirmed with a carbon monoxide reading in exhaled breath of < 10ppm) and a current, exclusive daily vaper, reporting e-cigarette use for ≥3 months. We have stipulated that current vapers should be using a nicotine strength e-liquid ≥12mg/mL (1.2 %) with a second or third generation device (open-systems with re-fillable tanks). Participants would be excluded from taking part if they report any of the following: Under 18 years of age; pregnant or lactating female; current tobacco smoking or a CO reading > 10ppm; marijuana or illicit drug use; are unable to provide written informed consent; or report any serious medical condition (including unstable angina, chronic obstructive pulmonary disease, cancer).

### Interventions

Table 1 illustrates participant interventions at all stages of the study.

**Table 1:** Provides a summary of participant and data collection timeline

	Study period							
	Enrolment	Allocation	Post-allocation					Close-out
Timepoint	*-t*_*1*_	0	*t*_*0*_	*t*_*1*_	*t*_*2*_	*t*_*3*_	*t*_*4*_	*t*_*x*_
Enrolment:								
Eligibility screen	X							
Informed consent	X							
Allocation		X						
Intervention:								
Fixed-user setting (6mg/mL nicotine or 18mg/mL)				X	X			
Fixed-user setting (6mg/mL nicotine or 18mg/mL)						X	X	
Assessments:								
Demographics (e.g. age, gender, ethnic group, educational qualification)	X		X					
Vaping and previous smoking history	X		X					
Breath carbon monoxide (CO) sample			X	X	X	X	X	
Puffing behaviour and participant reports				X	X	X	X	
Mood and Physical Symptoms Scale (MPSS) to record withdrawal symptoms			X	X	X	X	X	
Urge to vape			X	X	X	X	X	
Saliva sample			X	X	X	X	X	
Urine sample			X	X	X	X	X	
Adverse events			X	X	X	X	X	
Participant feedback								X
Debrief								X

There are two phases to this study. Phase one will measure puffing patterns in vapers over four weeks whilst they use high and low strength nicotine e-liquid under fixed and user-defined (changes to device allowed) settings, each for a week. The 4 conditions are: i) low strength (6mg/mL), fixed settings; ii) low strength user-defined settings; iii) high strength (18mg/mL) fixed settings; iv) high strength user-defined settings). Biomarkers of exposure will be measured at the end of each trial, in urine and saliva, for acrolein exposure (3-HPMA) and nicotine adsorption (cotinine) respectively. Phase two of this study will analyse e-cigarette aerosol (generated under average puffing regimes for each participant) in a laboratory setting.

Participants will be contacted upon expressing interest in taking part in the study, at this stage, the study details will be provided and the inclusion/exclusion criteria will be assessed. Upon agreeing to take part, participants will receive further details about the study, a consent form to complete and a date to attend the University for their baseline assessment.

At the baseline visit, the researcher will explain the study in further detail and read through the information sheet with the participant ensuring that s/he fully understands the requirements. Time will be allocated to answer any further questions before written informed consent is gained. Participants will then provide: a breath CO sample to ensure non-smoking status; a saliva and urine sample; and subjective ratings of nicotine craving and withdrawal symptoms. They will then be provided with a third generation device (eVic Supreme), nautilus tank and e-liquid (either 6mg/mL or 18mg/mL) to use for the next week and given the opportunity to practice using the device. Participants will also have the choice of one of four flavours of e-liquid to choose from, based on one popular flavour from different flavour categories (fruit, bakery, menthol, tobacco). Participants can sample the flavours at this initial meeting but are required to use the same e-liquid throughout the 4 week period.

Over the course of the 4 weeks, the e-cigarette will record puff number, puff duration, voltage, wattage and resistance. The device does not record puff velocity or volume, although these do not influence nicotine yield [20, 22]. Participants will be asked to refrain from using any other e-cigarette device, e-liquid or nicotine-containing products. We acknowledge that compliance may be an issue and will rely on participant’s self-reports regarding non-compliance, deleting data from non-compliant days. Participants will return to the University after one week to collect the next batch of e-liquid and to provide subjective ratings, breath CO, saliva and urine samples. The researcher will download puffing and device setting information from the device and the tank will be thoroughly cleaned and the atomiser replaced, participants will be asked to report on how much e-liquid they have consumed over the week. This procedure will be repeated on three further occasions in order to capture information on puffing patterns subjective effects ratings and nicotine intake and toxicants in urine under each of four conditions.

All participants will start on fixed device settings (to allow familiarisation with the device before allowing participants to change setting), but we aim to counterbalance the nicotine condition by half of the participants starting on high strength 18mg/mL and half on low strength 6mg/mL. At the beginning of week two, participants will continue to use the device under fixed-settings but will be given a different strength of nicotine e-liquid (dependent on which they received in week 1). We may, if necessary, offer participants a week’s break from the study during which time they can use their own device. If participant compliance and retention is an issue, we may recruit new participants for the second stage of phase one. In weeks 3 and 4, the counterbalancing of weeks 1 & 2 will be repeated under user-defined settings.

### Primary outcomes

#### Phase one

To document real-life puffing patterns (mean puff number, puff duration, IPI and mL of e-liquid consumed) and user behaviour (voltage, air-flow) associated with using high (18mg/mL) and low strength (6mg/mL) nicotine e-liquid illustrating if and how compensatory puffing behaviour occurs.

#### Phase two

To record toxicant/carcinogen emissions associated with puffing patterns/user behaviours associated with using low vs. high strength nicotine e-liquid as measured by biomarker levels in users’ urine and toxicant yields in e-cigarette aerosol.

### Secondary outcomes

#### Phase one

Subjective ratings of satisfaction, craving, withdrawal symptoms as well as nicotine intake (measured with cotinine and calculated via mL of e-liquid consumed) under the four conditions.

#### Phase two

A comparison of toxicant/carcinogen exposure levels associated with the puffing patterns/device settings used by our participants with those found in tobacco smoke and against recommended safety limits using existing published material.

### Participant timeline

Table 1 presents the participant timeline and associated interventions at each phase.

### Sample Size

Twenty regular vapers will be recruited. The proposed sample size is based on puff number and puff duration results from Dawkins et al., ([11]: 2016) [7 :*N =* 22;]. We observed effect sizes of d = 0.74 and d = 1.09 respectively. A sample of *N =* 14 for puff number and *N =* 11 for puff duration would allow us to detect effects at *p <* 0.10 with 90 % power.

### Recruitment

An advert will be placed on London South Bank University’s research participation scheme website, as well as advertisement in e-cigarette café’s and on e-cigarette forums as well as via twitter and direct e-mail to known vapers.

### Data collection

#### Phase one

Demographic information and vaping and previous smoking history will be collected via questionnaire at baseline assessment only.

At each session we will measure subjective craving (‘urge to vape’), and withdrawal symptoms through the use of the Mood and Physical Symptoms Scale [23] Direct (e.g. hit, satisfaction) and Adverse (headache, nausea) effects relating to nicotine/e-cigarette use will be measured as previously described in [19]. All questionnaires will be presented in paper format. No permission is required to use any of these scales.

E-cigarette puffing patterns and user behaviour will be recorded by retrieving information on puff number, puff duration, voltage, wattage and resistance recorded by a third generation electronic cigarette provided to participants at the baseline assessment. Air flow settings are not recorded and we rely on participant self-report for this information. Data will be downloaded from the participants’ device and screened using myVapors software.

At all assessment phases we will measure exhaled carbon monoxide using the Bedfont Micro Smokerlyzer to ensure non-smoking status. Participants producing readings above 10ppm will be excluded from the analysis.

Saliva and urine samples will be taken from each participant at every study visit (5 per participant). These will be posted to ABS laboratories within 48 h of collection where they will be frozen at −20°C and stored until the end of the study. Saliva will be assayed for cotinine to determine nicotine intake and 3-hydroxy cotinine to determine CYP 2A6 levels (rate of nicotine metabolism). This will allow us to determine how successful compensatory puffing behaviour has been, controlling for nicotine metabolism rate if necessary. Urine will be assayed for 3-HPMA to show acrolein exposure. If the development of an LC-MS/MS method for estimation of formaldehyde exposure that is being performed to support another study is successful, the urine samples will also be assayed for formaldehyde exposure.

#### Phase two

In order to explore toxicant and carcinogen emission associated with these puffing patterns, at the second phase of the study puffing and device setting information collected from the participants will be used to create average puffing regimes for each person. This phase of the study will involve no human participants.

Within this second phase we will use the same e-cigarette device and e-Liquids as phase 1. Aerosol will be generated using the automatic smoking simulator, Palaczbot (previously used in several published studies; [24–27]), a single, linear unit that allows the generation of e-cigarette aerosols under specific conditions allowing us to mimic the puffing behaviours of each participant in each condition. Carbonyl compounds will be quantified using AT 1200 liquid chromatograph (Agilent Technologies: as described below) and nicotine using Gas chromatography method with Thermionic Specific Detector (GC-TSD, Varian Inc.)

Aerosol will be generated 3 (or 6 if necessary) times for each analysis (for nicotine or carbonyl compounds) to replicate each participant’s puffing patterns using the same e-cigarette device setting under each of the four conditions in phase 1. Aerosol will be tested for formaldehyde, acetaldehyde, acrolein, benzaldehyde and nicotine emission with puffing regimes.

Carbonyl compounds (12 compounds): The method of aldehydes and ketones determination involves an adsorption of aldehydes and ketones aerosol mixture on a pipe filled with silica gel saturated with 2,4-dinitrophenylhydrazine, desorption of the compounds with acetonitrile in ultrasound washer, and determination using reverse phase technique of high performance liquid chromatography on AT 1200 liquid chromatograph (Agilent Technologies) equipped with Zorbax Eclipse PAH column (4,6 x 250 mm, 5μm) and spectrophotometric detector DAD. This allows determination of the following compounds: formaldehyde, acetaldehyde, acrolein, acetone, propionic aldehyde, crotonaldehyde, butanal, benzaldehyde, isovaleric aldehyde, valeric aldehyde, m-methylbenzaldehyde, o-methylbenzaldehyde, p-methylbenzaldehyde, hexanal, 2,5-dimethylbenzaldehyde [10].

Nicotine will be analysed using gas chromatography with Thermionic Specific Detector (GC-TSD, Varian Inc.). CP-Sil 8CB, 25 m × 0.25 mm × 0.39 mm (1.2 mm; Varian Inc.) capillary column with flow rate of helium of 2.4 ml/min will be used. Temperature of injector and detector will be 300 °C, column temperature will be increased from 60 to 200 °C (20 °C/min) and will be held for 5 min. Injection volume will be 1microlitre, and quinoline will be used as an internal standard.

### Statistical methods

For each participant in each of the four conditions, mean puff duration, puff number, and (for the user-defined conditions) wattage, voltage and air-flow for each day will be calculated and averaged across the seven days (or reduced for the number of compliant days if necessary) for that participant and condition. Repeated measures Analysis of Variance (ANOVA) will be used to compare puff duration, puff number, wattage, voltage, cotinine and acrolein across relevant conditions.

We will also present mean and standard deviations for carbonyl compounds and nicotine and use ANOVA to explore any effects of condition on these variables.

We will estimate health risks by comparing levels of toxicant and carcinogen exposure to a) tobacco smoking (documented in the literature) and b) the Health and Safety Executive (HSE) workplace exposure limits [28] in order to provide useful information for public health professionals and policy makers about the relative harms associated with this level of exposure.

For each toxicant/carcinogen analysed, we will calculate a new exposure index (e-cigarette exposure index, EEI), i.e. total doses inhaled by individuals from e-cigarettes over one day. The EEI for each toxicant will be calculated by comparing doses inhaled from e-cigarette to doses that would be inhaled from air with toxicant concentration specified by the HSE long-term exposure limits. We will assume an average lung ventilation rate and take into account number of puffs taken per day. The EEI index may be useful to compare relative risk of inhaling toxicants from e-cigarettes. EEIs higher than 1.0 would indicate that mean exposures from e-cigarettes exceed exposure defined by HSE standards. We will verify if there is a correlation between the calculated EEI generated from the aerosol analysis with 3-HMPA concentration and (if assay is successful) formaldehyde estimated in urine. The higher EEI, the higher health risk for users associated with inhaling specific toxicants.

### Ethical considerations

Ethical approval was granted by London South Bank University (Application reference: UREC 1604) and has been funded by Cancer Research UK (Application reference: C50878/A21130). Informed consent will be collected in writing at the baseline session at the University prior to any data collection. Participants will have already received the information sheet and consent form via e-mail and had a chance to discuss any aspect of the study via e-mail or over the telephone with the researcher.

No sensitive data will be transferred electronically; it will also be anonymised beforehand (i.e. numerical codes will be used rather than names).

## Discussion

The aim of this research is to investigate real-life e-cigarette puffing patterns and user behaviour associated with high and low strength nicotine e-liquids and how this affects toxicant and carcinogen exposure.

There are notable factors which will challenge the process of gaining the necessary evidence. The first relates to recruitment and retention. We are seeking experienced vapers with >3 months experience; we may expect vapers with such experience to be attached to their current devices, settings and e-liquid strengths and flavours and therefore not forthcoming in sampling new products and e-liquids. In order to mitigate this we have budgeted for wide advertisement of the study and allowed 7 months for recruitment and complete testing of 20 participants. Furthermore, we wish to measure long-term puffing patterns (over 4 weeks) beyond the timescale of previous research [8, 11, 29, 30]. For the same reasons highlighted above, participants may not be fully compliant, and may quit during our trial or (especially during the low nicotine strength weeks) compensate with their own devices or with cigarette smoking. We seek to overcome this by a) discussing flavour requirements and offering a range of e-liquid flavours, b) encouraging contact with the research team throughout the study c) emphasising honest reporting about non-compliance/use of other nicotine-containing products and d) recruiting exclusive vapers (non dual-users) who haven’t smoked for at least 3 months. A seven day testing period under each condition means that we can exclude data from non-compliant days without it having a significant impact on our primary outcome, puffing patterns (puff number, duration, IPI and mL e-liquid consumed), which is essential for phase two. 3-HMPA (for acrolein exposure) and cotinine, with a half-life of 72 h and averaging 17 h respectively, [31, 32] may be more susceptible to the use of other nicotine-containing products (especially to occasional tobacco cigarette smoking) but will we still be able to measure these in aerosol in phase two if non-compliance proves to be an issue. Finally, non-compliance itself is an interesting outcome and will still allow us to conduct meaningful analysis of toxicant/carcinogen exposure associated with different puffing regimes.

As in our laboratory study [11], we will use inbuilt e-cigarette software to capture information on puffing patterns, a straightforward and arguably, more ecologically valid procedure than CReSS pocket devices. This will provide us with detailed information on each puff (time of puff, length of puff), the IPI and overall number of puffs allowing us to exclude data from non-compliant days and to conduct more fine-grained analysis on the nature of puffing patterns (timings, cycles and episodes of puffs over 24 h periods) if necessary. However, the device does not record puff velocity or volume, although as noted earlier these parameters do not appear to influence nicotine yield [20, 22]. In the user-defined conditions, we will allow users to adjust the voltage (recorded for each puff by the device) and air-flow. The latter is not captured by the inbuilt software and we will therefore have to rely on participant self-report (average for the day) for this information. Experienced users may also be accustomed to adjusting the resistance of the atomiser/coil when switching to a lower nicotine strength e-liquid. We will request that participants do not change the atomiser on the device but if they do, the inbuilt software will record this information.

Although our study will not provide a definitive answer to the safety of e-cigarette use under all circumstances, it will help to inform public health messaging and policy making regarding the ‘safest’ way to vape and the regulation of nicotine e-liquid strengths. If compensatory puffing patterns associated with using lower nicotine strength e-liquids result in higher toxicant exposure, the use of higher nicotine strength e-liquids may be recommended. The findings will also provide the foundations for future studies exploring the effects of a broader range of e-cigarette devices, settings, nicotine strengths and flavourings on puffing patterns and help to inform the parameters of future aerosol toxicology studies.

The data collected are intended to benefit the general public; we will therefore preserve all data resulting from the study (with the exception of personal data) and make it publically available with as few restrictions as possible. We will disseminate a lay summary for participants, explaining our findings and their importance. Once the data has been finalized, it will be deposited in London South Bank University’s open data repository. The findings will be disseminated via open access peer-review publication, conference presentations and press releases, and shared with a number of charities, practitioners and public health and policy organisations via presentations, briefing papers and web-based material.

## Abbreviations

3-HPMA: 3-hydroxypropylmercapturic acid
EEI: Estimated Exposure Index
IPI: Inter-puff interval
LC-MS/MS: Liquid chromatography–mass spectrometry
